# Impact of Dietary Protein Concentration and Quality on Immune Function of Cats

**DOI:** 10.1371/journal.pone.0169822

**Published:** 2017-01-10

**Authors:** Nadine Paßlack, Barbara Kohn, Marcus G. Doherr, Jürgen Zentek

**Affiliations:** 1 Institute of Animal Nutrition, Department of Veterinary Medicine, Freie Universität Berlin, Berlin, Germany; 2 Clinic of Small Animals, Department of Veterinary Medicine, Freie Universität Berlin, Oertzenweg 19b, Berlin, Germany; 3 Institute of Veterinary Epidemiology and Biostatistics, Department of Veterinary Medicine, Freie Universität Berlin, Königsweg, Berlin, Germany; Colorado State University, UNITED STATES

## Abstract

Protein levels and quality in cat food can vary significantly and might affect immune function in various ways. In the present study, 3 diets with a low protein quality (LQ) and 3 diets with a high protein quality (HQ) were offered to 10 healthy adult cats for 6 weeks each, using a randomized cross-over design. The LQ and HQ diets differed in the collagen content and had low (36.7% and 36.2%), medium (45.0% and 43.3%) and high (56.1% and 54.9%) protein levels. At the end of each feeding period, blood was collected for phenotyping of leukocyte subsets, lymphocyte proliferation assay and cytokine measurements, phagocytosis assay and differential blood count. The results demonstrated no group differences for numbers of CD4^+^CD8^-^, CD4^+^CD8^+^, CD4^-^CD8^+^, MHCII^+^, CD21^+^, SWC3^+^ and CD14^+^ cells in the blood of the cats. Proliferative activity of lymphocytes when stimulated with pokeweed mitogen, Concanavalin A and Phytohemagglutinin, M form did not differ depending on the dietary protein concentration and quality. Concentrations of tumor necrosis factor alpha and interferon gamma in the supernatant of the proliferation assay were also not affected by the dietary treatment. Blood monocyte phagocytic activity was higher (*P* = 0.048) and cell numbers of eosinophilic granulocytes in the blood were lower (*P* = 0.047) when cats were fed the low protein diets. In conclusion, only a few differences in feline immune cell populations and activity depending on dietary protein supply could be detected. However, the observed increase of eosinophilic granulocytes by a higher protein intake indicates an activation of immunological mechanisms and requires further investigation.

## Introduction

Dietary protein and amino acids are important for the function of the immune system and certain amino acids, such as arginine, can also act as immunomodulators in the organism [1, 2]. The role of dietary protein in immune function in cats is of particular interest in the course of allergic reactions. It is well known that food allergens are usually proteins with a high molecular weight, and that hypersensitivity reactions can include humoral and cellular mechanisms [3]. In healthy cats, however, the relevance of protein intake for immune function has scarcely been investigated to date. Cave and Marks [4] evaluated whether there is a difference in immunogenicity between unprocessed dietary protein and protein after retort processing (canning) in young cats. The authors could not detect group differences in serum immunoglobulin (Ig)G and IgM or in proliferative activity of lymphocytes from mesenteric lymph nodes. However, a salivary IgA response was observed when cats received processed casein, but not in cats fed unprocessed casein [4]. Therefore, a difference in immunogenicity of certain proteins which was dependent on heat processing was assumed [4].

Considering that protein quality and concentrations in commercial diets for cats can show significant variations, differences in amino acid supply may be important. Specific amino acids can affect immune function by several mechanisms [1, 2]. For instance, it has been demonstrated that the addition of 1.9% arginine to a low protein canned diet led to a greater phagocyte function and an increased proliferative activity of T cells in response to the mitogen Phytohaemagglutanin in healthy adult cats [5]. Nevertheless, detailed studies on the effect of variations in protein intake on immunological parameters in cats are lacking. The aim of the present study was to evaluate the effect of protein quality and concentration in a canned diet for cats on the composition and activity of immune cell populations in the blood of adult cats. It was hypothesised that changes in protein intake and quality would affect feline immune function on a cellular basis in the healthy organism.

## Methods

### Study design

Ten healthy adult research cats (European shorthair; 6.8 ± 1.5 years old) from the Institute of Animal Nutrition, Freie Universität Berlin, were used for this study. The cats were housed in the facilities (approx. 24 m^2^) of the Institute with a constant temperature (21°C) and light (12 hours light, 12 hours darkness) regimen. The animal care and use protocol was approved by the Animal Welfare Committee (LaGeSo, Berlin, Germany, G 0138/12). At the end of this study, the cats remained in the Institute for subsequent investigations.

Three diets with a high protein quality (36.2%, 43.3% and 54.9% crude protein (CP) in dry matter (DM)) and three diets with a low protein quality (36.7%, 45.0% and 56.1% CP in DM) were fed for six weeks each, using a randomized cross-over design. During each six-week feeding period, cats were housed in groups for the first 31 days, followed by a 2x four-day individual housing in metabolic cages with a three-day group housing (rest period) between. Individual housing was required for urine and faeces collection from the cats (data are not part of this publication). The floor area of the metabolic cages was 0.74–0.97 m^2^.

A high dietary protein quality was achieved by the use of higher amounts of meat and blood meal than in the low protein quality diets. The diets with the lower protein quality contained higher amounts of collagen-rich material (trachea and greaves meal). In order to achieve a comparable energy density among all diets, amounts of sunflower oil in the diets were adjusted (approximately 4.6% oil in the low protein level diets and 3.0% oil in the high protein level diets). Feed allowances were calculated in order to maintain body weight (BW) of the cats [6]. The cats were fed individually throughout the study, and feed intake was recorded daily.

Blood was collected from fasted cats at the end of each feeding period. Blood was collected in potassium EDTA tubes (Micro tube K3E, Sarstedt, Nümbrecht, Germany) and lithium heparin tubes (S-Monovette^®^, Sarstedt, Nümbrecht, Germany) by routine cephalic venipuncture.

### Analyses of the experimental diets

Results of the analyses of the experimental diets are presented in Tables 1 and 2. Concentrations of crude nutrients were determined according to the Weende method [7]. Analysis of crude fat was modified [8]. In short, fat extraction was carried out with petroleum diethyl ether for 3 hours. After vaporisation in a compartment dryer, the samples were cooled in a desiccator and the amount of dietary crude fat was calculated. Mineral analyses were carried out as described elsewhere [9].

**Table 1:** Nutrient analysis of the experimental diets^1^^,^^2^.

Analyzed	LQ_low_	LQ_medium_	LQ_high_	HQ_low_	HQ_medium_	HQ_high_
Dry matter (g/kg)	200	189	204	216	203	193
**In g/kg dry matter**						
Crude protein	367	450	561	362	433	549
Crude fat	284	296	271	294	318	262
Crude fiber	3.14	7.05	10.5	6.75	4.46	6.78
Crude ash	77.2	73.8	83.4	68.3	68.9	75.2
Ca	11.2	11.4	12.6	10.7	11.2	11.8
P	8.79	8.34	8.88	7.73	8.23	8.71
Na	5.83	5.75	8.20	4.88	5.78	6.74
K	11.1	9.35	12.4	8.75	8.58	8.67
Mg	0.48	0.48	0.47	0.45	0.47	0.49
Metabolizable energy (MJ/kg dry matter)^3^	20.4	20.8	20.4	20.6	21.3	20.4

^1^Ingredients in descending order (low protein quality diets): liver, trachea, wheat flour, meat, sunflower oil, greaves meal, blood meal, minerals, gelling and thickening agents, vitamins.

^2^Ingredients in descending order (high protein quality diets): liver, meat, wheat flour, sunflower oil, blood meal, greaves meal, minerals, gelling and thickening agents, vitamins.

^3^Calculated according to NRC [6].

**Table 2:** Amino acid concentrations (g/kg dry matter) in the experimental diets.

	LQ_low_	LQ_medium_	LQ_high_	HQ_low_	HQ_medium_	HQ_high_
Aspartic acid	26.5	33.3	38.7	25.1	33.8	42.9
Threonine	14.2	17.8	20.2	14.1	18.3	23.6
Serine	14.6	18.9	21.9	14.8	18.5	23.2
Glutamic acid	35.9	40.0	49.0	35.7	38.9	44.1
Glycine	36.0	39.1	52.6	28.0	23.8	36.6
Alanine	18.9	26.6	32.9	17.6	23.1	30.3
Valine	18.0	22.4	25.9	12.2	22.4	27.0
Isoleucine	12.8	16.1	17.8	9.2	17.2	21.2
Leucine	24.7	31.4	37.6	24.4	32.4	39.3
Tyrosine	10.4	13.8	16.1	10.6	14.9	18.7
Phenylalanine	13.9	18.3	21.6	14.1	18.8	24.1
Histidine	12.0	15.4	15.5	10.9	17.1	20.8
Lysine	21.6	28.3	33.6	20.9	29.6	36.5
Arginine	17.3	24.9	30.5	15.4	23.6	31.2
Proline	16.1	25.0	30.3	15.9	17.3	23.4
Methionine	6.29	8.77	10.1	5.74	9.86	11.6
Cysteine	6.47	7.67	8.93	6.63	8.75	10.4
Hydroxyproline	3.76	8.45	9.44	2.56	3.76	4.45
Taurine	5.32	4.36	3.90	4.13	3.81	3.85

For analysis of amino acids, diets were ground to a particle size of 0.5 mm. Sample preparation for the measurement of methionine and cysteine included an oxidation and hydrolysis step, whereas samples were only hydrolysed for the measurement of other amino acids. For oxidation, an oxidation solution (0.5 ml H_2_O_2_ (30%), 4.5 ml formic acid (85%), 25μl phenol) was prepared and placed into a water bath at 30°C for 1 hour. After cooling in an ice bath for 5 minutes, 5 ml of the solution were mixed with 500 mg of the samples. The samples were placed into an ice bath and incubated in a refrigerator for 24 hours. Subsequently, 0.9 g sodium metabisulphite was added to each sample, followed by the hydrolysis step. Therefore, 500 mg sample was mixed with 25 ml hydrochloric acid (6 molar) in a 100 ml glass bottle (DURAN Group GmbH, Mainz, Germany). The bottle was placed into a compartment dryer (110°C) for 1 hour. After sealing the bottle, the samples were incubated in the compartment dryer for a further 23 hours. The samples were air-dried, placed into an ice bath, and 20 ml of sodium hydroxide solution (7.5 mol/l) was added slowly. Subsequently, hydrochloric acid or sodium hydroxide were added in order to achieve a pH of 2.20. This solution was transferred into 100 ml graduated flasks and made up to volume using sodium loading buffer pH 2.20 (Biochrom Ltd, Cambridge, UK). Subsequently, 1.5 ml of the samples were transferred by a membrane filter (syringe filter with cellulose acetate membrane, diameter: 25 mm, pore size: 0.45 μm; VWR International GmbH, Darmstadt, Germany) into vials. Amino acid concentrations in the samples were determined by ion chromatography (Biochrom 20 Plus, Amersham Pharmacia Biotech, Piscataway, USA), where a lithium column (High Performance) was used (Biochrom Ltd), following standard procedures (Laborservice Onken GmbH, Gründau, Germany). For calibration, a standard “feedstuff hydrolysate” (order no. 5.403.154; Laborservice Onken GmbH, Gründau, Germany) was used.

### Differential blood count

A differential cell count (Hemomat-K, Biomed, Oberschleißheim, Germany; Pappenheim stain) was performed on potassium EDTA (K-EDTA)-anticoagulated whole blood using standard laboratory techniques.

### Phenotyping

20 μl heparin blood were either mixed with 25 μl phosphate buffered saline (PBS; Biochrom GmbH, Berlin, Germany) (control) or with 25 μl primary antibody. The following primary antibodies and dilution were used: mouse anti-cat CD4:FITC (Serotech; MCA1346F; 1:20 dilution), mouse anti-cat CD8ALPHA/BETA (Serotech; MCA1347PE; 1:20 dilution), mouse anti-canine CD21 (Serotech; MCA1781S; 1:100 dilution), mouse anti-human CD14: FITC (Serotech; MCA1568F; 1:20 dilution), mouse anti-cat MHC Class II (Serotech; MCA2723; 1:20 dilution) and P-DH59B, Specificity CD172a (Monoclonal Antibody Center; P-BOV2049; 1:100 dilution). The primary antibodies were diluted with PBS. According to the manufacturer, the mouse anti-canine CD21 and mouse anti-human CD14: FITC antibodies show reactivity for cats, which was validated in the Institute prior to the beginning of the study.

The samples were incubated at room temperature for 15 minutes with light protection. Subsequently, 2 ml PBS were added to the samples and the mixture was centrifuged for 5 minutes at 389 x g and 4°C. The supernatant was discarded and 50 μl PBS (control and samples with mouse anti-cat CD4:FITC and mouse anti-cat CD8ALPHA/BETA) or 50 μl of the secondary antibody (other samples; Goat F(ab’)2 anti-mouse IgG1-RPE, Human adsorbed, Southern-Biotech, 1072–09; Goat anti-mouse IgG2b-RPE, Human adsorbed, Southern-Biotech, 1090–09) was added to the samples. After incubation for 15 minutes at room temperature and light protection, 900 μl distilled water (Aqua bidest.) were added to the samples and vortexed three times for 3 seconds each. Subsequently, 100 μl 10x PBS were added. The samples were vortexed and centrifuged for 5 minutes at 389 x g and 4°C. The supernatant was discarded, the samples were vortexed and 200 μl FACS buffer (1x PBS, 0.5% bovine serum albumin) were added to each sample. For the measurements, the flow cytometer MACSQuant (Miltenyi Biotec GmbH, Bergisch Gladbach, Germany) was used. At least 10,000 events within the respective leukocyte gate were collected in each sample for the analyses.

### Proliferative activity

Peripheral blood mononuclear cells (PBMC) were isolated from fresh blood under sterile conditions. Blood was diluted (1:2) with PBS. This mixture was carefully pipetted on 3 ml of Ficoll (Biochrom GmbH) in a 15 ml tube and centrifuged at 500 x g and room temperature for 30 minutes (no/reduced brake). The buffy coat was pipetted into a new 15 ml tube. After 10 ml of cold PBS was added, the samples were centrifuged for 10 minutes at 400 x g and 4°C. The supernatant was discarded and 10 ml of cold PBS were added to the samples. Subsequently, the samples were centrifuged for 10 minutes at 300 x g and 4°C. The supernatant was discarded and the pellet was resuspended with 1 ml of cold cell culture media (RPMI 1640 + 10% fetal calf serum + 1% penicillin/streptomycin; all Biochrom GmbH). Cells were counted, and a cell density of 4 x 10^6^/ml was prepared for the following proliferation assay. Cell culture media was used for this preparation.

For the proliferation assay, 96 well plates, round bottom, suitable for cell or tissue culture (Greiner, #650180) were used. Twenty-five μl mitogens and 100 μl (4x10^5^) cells were pipetted into each well. For negative control, 25 μl cell culture media was used in place of the mitogen. The concentration of the mitogens (all Sigma-Aldrich Chemie GmbH, Munich, Germany) in the wells was 2.5 μg/ml for pokeweed mitogen (PWM), 5 μg/ml for Concanavalin A (Con A) and 10 μg/ml for Phytohemagglutinin, M form (PHA-M). The plates were placed into a CO_2_ incubator (37°C, 5% CO_2_; Biocenter BC170, SalvisLab, Rotkreuz, Switzerland) for 48 hours. After the incubation, 12.5 μl of 5-Bromo-2-Deoxyuridine (BrdU; Sigma-Aldrich Chemie GmbH) was pipetted into each well. The concentration of BrdU was 60 μM/well. The plates were placed into the CO_2_ incubator for 24 hours. After this incubation, the following steps were performed under non-sterile conditions. Cells were pipetted into 3.5 ml round bottom tubes (VWR International GmbH, Darmstadt, Germany), where the wells containing the same mitogen were pooled for each animal. The tubes were centrifuged for 5 minutes at 389 x g and 4°C. The supernatant was used for the following cytokine measurements (see below) and the pellet was resuspended with 500 μl BD FACS Perm 2 buffer (BD, Heidelberg, Germany; diluted 1:10 with Aqua dest). The tubes were incubated on ice at 4°C for at least 18 hours. After this incubation, 2 ml of FACS buffer was added to each tube. The tubes were centrifuged for 5 minutes at 389 x g and 4°C. The supernatant was discarded and the pellet was resuspended with 250 μl of DNAse I from bovine pancreas (1 mg/ml) (Sigma-Aldrich Chemie GmbH). After incubation at 37°C for 30 minutes, 2 ml FACS buffer was added to each tube, and the tubes were centrifuged for 5 minutes at 389 x g and 4°C. The supernatant was discarded and the pellet was resuspended with 50 μl of anti-BrdU FITC antibody (BD Pharmingen, #556028; diluted 1:5 with FACS buffer). After incubation for 30 minutes on ice and with light protection, 2 ml of FACS buffer was added to each tube. The tubes were centrifuged for 5 minutes at 389 x g and 4°C. The supernatant was discarded, and the pellet was resuspended with 300 μl FACS buffer. Subsequently, the measurement was performed using the flow cytometer MACSQuant (Miltenyi Biotec GmbH). At least 10,000 events/sample were collected within the lymphocyte gate. Relative proliferation index (%) was calculated from the proliferative activity of stimulated lymphocytes (% gated) divided by the proliferative activity of unstimulated lymphocytes (% gated).

### Phagocytic activity

The phagocytic activity of the monocytes and granulocytes in the blood of the cats was measured using the commercial PHAGOTEST^TM^ (Glycotope Biotechnology GmbH, Heidelberg, Germany). Sample preparation and measurements were performed according to the instructions of the manufacturer. For the measurements, the flow cytometer MACSQuant (Miltenyi Biotec GmbH) was used, and at least 10,000 leukocytes/sample were collected for the analyses.

### Cytokine secretion

Concentrations of tumor necrosis factor alpha (TNF-α) and interferon gamma (IFN-γ) were measured in the cell-free supernatant of the proliferation assay. Commercial Enzyme Linked Immunosorbent Assays (ELISA) were used and assays performed as indicated by the manufacturer (R&D Systems GmbH, Wiesbaden-Nordenstadt, Germany).

### Statistical analysis

For statistical data analysis, SPSS 22 (SPSS Inc., Chicago, Illinois, USA) was used. For each parameter, a repeated-measures ANOVA was performed (within-subject factors: dietary protein level (3) and dietary protein quality (2)). When the interaction between dietary protein level and dietary protein quality was significant, linear and quadratic polynomial contrasts were calculated separately for protein level in the high protein quality groups and for protein level in the low protein quality groups. In case of a non-significant interaction between dietary protein level and dietary protein quality, the high and low dietary protein quality groups were examined together at the same dietary protein levels, and linear and quadratic polynomial contrasts for dietary protein level and linear polynomial contrasts for dietary protein quality were calculated. For the data analysis on cytokine secretion of the lymphocytes, one-factor analysis of variance (fixed factor: diet) and Scheffe´ (variance equality) or Tamhane 2 (variance inequality) post hoc tests were used. Data are presented in Tables as means and standard errors. The level of significance was *P* < 0.05. As one cat with health problems was replaced in the first third of the study (see results), group differences were only calculated with the data set of the compensatory cat (diets LQ_low_, HQ_low_, HQ_medium_ and HQ_high_).

## Results

### Animal health

One cat showed health problems which were not related to the feeding study. This cat was replaced by another cat with a comparable body weight and age in the first third of the study.

### Differential blood count

Percentage of eosinophilic granulocytes was lower when the low protein diets were fed, independently of the dietary protein quality (linear contrast for protein level: *P* = 0.047) (Table 3). Percentages of lymphocytes, neutrophilic and basophilic granulocytes and monocytes were not affected by the dietary protein level or quality.

**Table 3:** Differential blood count of cats fed diets with varying protein concentrations and qualities. Mean and pooled SEM.

								*P* values (polynomial contrasts)							
	LQ_low_(n = 10)	LQ_medium_(n = 9)	LQ_high_(n = 9)	HQ_low_(n = 10)	HQ_medium_(n = 10)	HQ_high_(n = 10)	SEM	Inter-action	LQ		HQ		Protein level		Protein quality
									Lin.	Quadr.	Lin.	Quadr.	Lin.	Quadr.	
**In %**															
Neutrophilic granulocytes	65.3	62.2	59.8	64.3	66.2	60.8	0.99	0.369	-	-	-	-	0.066	0.363	0.218
Lympho-cytes	27.8	29.7	31.7	27.5	25.3	31.2	0.99	0.358	-	-	-	-	0.197	0.301	0.205
Monocytes	2.32	2.32	2.92	2.55	2.46	2.52	0.12	0.150	-	-	-	-	0.063	0.379	0.270
Eosinophilic granulocytes	4.58	5.67	5.46	5.42	5.92	5.34	0.37	0.637	-	-	-	-	**0.047**	0.396	0.982
Basophilic granulocytes	0.07	0.09	0.09	0.21	0.08	0.11	0.02	0.171	-	-	-	-	0.417	0.134	0.234

### Phenotyping

Numbers (% gated) of T-helper cells (CD4^+^CD8^-^; CD4^+^CD8^+^), cytolytic T cells (CD4^-^CD8^+^), antigen-presenting cells (MHCII^+^), myeloid cells (SWC3^+^ and CD14^+^) and B cells (CD21^+^) did not differ among the dietary treatment groups (*P* > 0.05). Means between 31.3–34.1% (SEM: 1.25%) (CD4^+^), 19.2–20.9% (SEM: 0.79%) (CD8^+^), 1.17–2.88% (SEM: 0.28%) (CD4^+^CD8^+^), 8.08–9.84% (SEM: 0.63%) (CD14^+^), 22.8–27.0% (SEM: 1.23%) (CD21^+^), 87.6–90.4% (SEM: 0.57%) (MHCII^+^) and 94.4–98.1% (SEM: 0.47%) were observed among the treatment groups.

### Proliferative activity

Proliferative activity of lymphocytes after stimulation with the mitogens PWM, ConA and PHA-M was not affected by a varying protein level or quality in the diets (Table 4).

**Table 4:** Proliferative activity of lymphocytes of cats fed diets with varying protein concentrations and qualities. Mean and pooled SEM. Proliferation index was calculated as the proliferative activity of stimulated lymphocytes (% gated) divided by the proliferative activity of unstimulated lymphocytes (% gated).

								*P* values (polynomial contrasts)							
	LQ_low_(n = 10)	LQ_medium_(n = 9)	LQ_high_(n = 9)	HQ_low_(n = 10)	HQ_medium_(n = 10)	HQ_high_(n = 10)	SEM	Inter-action	LQ		HQ		Protein level		Protein quality
									Lin.	Quadr.	Lin.	Quadr.	Lin.	Quadr.	
PWM	28.6	17.7	9.08	17.5	21.2	24.7	3.84	0.367	-	-	-	-	0.485	0.953	0.739
ConA	27.1	16.3	11.5	29.4	28.9	42.2	5.07	0.413	-	**-**	-	-	0.934	0.686	0.265
PHA-M	2.85	1.90	2.59	2.71	2.91	2.87	0.32	0.674	-	-	-	-	0.919	0.651	0.392

PWM: pokeweed mitogen; ConA: Concanavalin A; PHA-M: Phytohemagglutinin, M form.

### Phagocytic activity

Monocytes internalized a greater number of bacteria when cats received the low protein level diets (linear contrast for protein level: *P* = 0.048), while the number of phagocytic monocytes did not differ depending on the dietary treatment (Table 5). Phagocytic activity of granulocytes was not affected by a varying dietary protein concentration or quality.

**Table 5:** Phagocytic activity of monocytes and granulocytes of cats fed diets with varying protein concentrations and qualities. Mean and pooled SEM.

								*P* values (polynomial contrasts)							
	LQ_low_(n = 10)	LQ_medium_(n = 9)	LQ_high_(n = 9)	HQ_low_(n = 10)	HQ_medium_(n = 10)	HQ_high_(n = 10)	SEM	Inter-action	LQ		HQ		Protein level		Protein quality
									Lin.	Quadr.	Lin.	Quadr.	Lin.	Quadr.	
**% gated**															
Phagocytic monocytes	76.3	73.0	79.8	76.4	67.0	70.2	1.75	0.668	-	-	-	-	0.678	0.139	0.228
Phagocytic granulocytes	94.2	89.3	95.0	94.2	91.8	91.8	0.75	0.552	-	-	-	-	0.504	0.103	0.247
**Number of bacteria**															
Per monocyte	64.8	58.9	54.8	63.3	52.7	53.6	2.08	0.936	-	-	-	-	**0.048**	**0.032**	0.669
Per granulocyte	89.9	76.0	78.5	87.1	82.8	87.0	4.20	0.666	-	-	-	-	0.868	0.244	0.845

### Cytokine secretion

Concentrations of TNF-α and IFN-γ in the supernatant of the proliferation assays did not differ depending on the dietary treatment of the cats (*P* > 0.05). Means for TNF-α secretion from unstimulated lymphocytes ranged between 48.9–66.2 pg/ml (SEM: 6.29 pg/ml), from lymphocytes stimulated with PWM between 81.5–141 pg/ml (SEM: 9.37 pg/ml), from lymphocytes stimulated with ConA between 152–396 pg/ml (SEM: 58.1 pg/ml) and from lymphocytes stimulated with PHA-M between 33.6–53.1 pg/ml (SEM: 3.62 pg/ml). For IFN-γ secretion, mean values between 544–1191 pg/ml (SEM: 187 pg/ml; unstimulated lymphocytes), 4080–7049 pg/ml (SEM: 576 pg/ml; lymphocytes stimulated with PWM), 3116–7604 pg/ml (SEM: 611 pg/ml; lymphocytes stimulated with ConA) and 297–537 pg/ml (SEM: 39.8 pg/ml; lymphocytes stimulated with PHA-M) were detected.

## Discussion

Protein sources in cat food can vary in terms of quality and quantity. Changes in protein or amino acid intake and also digestibility of protein might affect immunological functions of the organisms in various ways. For instance, specific amino acids have been demonstrated to stimulate immune function [1]. On the other hand, a lower dietary protein quality might be related to a higher microbial fermentation of undigested protein in the large intestine [10] with resulting changes in microbial composition and activity which could in turn affect the immune system by several mechanisms [11]. In particular, microbial fermentation products might have a direct impact on immune cell function or, as an indirect effect, may influence the epithelial integrity of the gut and therefore exposure of the immune system to dietary antigens [11].

The composition of the experimental diets used in the present study was chosen to reflect significant variations in dietary protein level and quality. Crude protein concentrations differed by 50% between the low and high protein diets. Changes in dietary protein quality were achieved by varying amounts of collagen-rich ingredients, and differences in hydroxyproline concentrations, an amino acid found in high amounts in collagen tissue, were approximately doubled in the low dietary protein quality diets when compared to the diets with the high protein quality. Nevertheless, all diets fulfilled the recommended allowance of 200 g crude protein/kg dry matter for cats [6]. As the present study aimed to evaluate the effect of changes in dietary protein supply on the feline immune system under physiological conditions, no protein or amino acid deprivation was induced, and only healthy cats were considered for this investigation. In this context, it should also be considered that the present study did not measure amino acid concentrations in the blood of the animals. The detected effects of the dietary protein level on immune parameters in the cats can therefore be interpreted only from a quantitative point of view.

The present results demonstrated that varying protein supply had limited effects on immune function in healthy adult cats. Phagocytic activity of monocytes was lower when high protein diets were fed. Interestingly, this effect was observed in the low and high dietary protein quality groups. It can be hypothesized that it was not the lower protein level which led to the higher phagocytic activity of the feline monocytes but dietary factors other than protein. In particular, fatty acids might be considered. In order to achieve a comparable energy density in the experimental diets, concentrations of sunflower oil were adjusted among the diets. The low protein diets contained higher amounts of oil (approximately 4.6%) compared to the diets with the moderate and high protein levels (approximately 3.5% and 3.0% sunflower oil). A higher intake of fatty acids might have stimulated the phagocytic activity of the monocytes in the blood of the cats when the low protein level diets were fed. In this context, previous investigations have demonstrated that conjugated linoleic acids increased phagocytosis of macrophages in different animal species [12–14]. Future studies should investigate the effects of fatty acids on phagocytic activity of feline monocytes in more detail to determine if this could be an explanation for the observed group differences in the present study.

A linear effect on percentage of eosinophilic granulocytes in the blood of the cats was demonstrated in the present investigation, where the lowest percentage was observed in the low protein level groups. Eosinophilic granulocytes are involved in several inflammatory processes and can modulate immunological responses [15]. Eosinophilic granulocytes are particularly associated with allergic reactions [15]. In cats, eosinophilia is observed in 20–50% of animals with food allergy [16, 17]. Considering that allergic responses are usually mediated by proteins [3], the higher percentage of eosinophilic granulocytes in the higher dietary protein level groups may represent an activation of the feline immune system by a higher protein intake. However, it should be considered that percentages of eosinophils in the blood of all cats were within the normal range and therefore do not indicate a pathological situation. Nevertheless, as the described linear effect of dietary protein level on feline blood eosinophils was observed both, in the high and low dietary protein quality groups, a general immunostimulating effect, although not disease-associated, of a higher protein intake might be assumed based on the present results.

There were no diet-dependent changes in TNF-α and IFN-γ concentrations to correlate with the change in eosinophil granulocyte cell numbers. Although an activating effect of these cytokines on eosinophil function is assumed, further stimulating factors are relevant for the development and activation of these cells [15]. In particular, eosinophil surface receptors for cytokines, complement and immunoglobulins can be involved [15]. Therefore, future studies should investigate the relationship between the increase in blood eosinophils and high dietary protein levels.

It should finally be noticed that the experimental diets of the present study showed some variations in fibre content, particularly with regard to the low protein quality diets. In general, high dietary fibre concentrations can lower bioavailability of amino acids [18]. However, quantitative protein supply markedly increased from the low to the high protein level diets, and group differences were only detected for the dietary protein concentration, independently of the dietary protein quality. Thus, the possible impact of the varying dietary fibre content, particularly among the low protein quality diets, on amino acid bioavailability might be of low importance. However, as bioavailability was not estimated, particularly by measuring blood levels of amino acids, the variations in dietary fibre concentration require a careful interpretation of the present data.

## Conclusion

In conclusion, variations in dietary protein concentration and quality had only minor impact on immune function of healthy adult cats. A higher phagocytic activity of blood monocytes observed in the low dietary protein level groups might possibly be attributed to dietary factors other than protein, particularly with regard to the higher amounts of fatty acids in these low protein diets. The detected lower number of eosinophilic granulocytes when diets with a lower protein level were fed indicates an activation of immunological mechanisms in response to a higher protein intake.
